# Risk factors of colistin safety according to administration routes: Intravenous and aerosolized colistin

**DOI:** 10.1371/journal.pone.0207588

**Published:** 2018-11-21

**Authors:** Kyoung Lok Min, Eun Sun Son, Jae Song Kim, Soo Hyun Kim, Sun Mi Jung, Min Jung Chang

**Affiliations:** 1 Department of Pharmaceutical Medicine and Regulatory Sciences, Colleges of Medicine and Pharmacy, Yonsei University, Incheon, Republic of Korea; 2 Department of Pharmacy, Severance Hospital, Yonsei University Health System, Seoul, Republic of Korea; 3 Department of Pharmacy and Yonsei Institute of Pharmaceutical Sciences, College of Pharmacy, Yonsei University, Incheon, Republic of Korea; 4 Department of Industrial Pharmaceutical Science, Colleges of Pharmacy, Yonsei University, Incheon, Republic of Korea; IRCCS Policlinico S.Donato, ITALY

## Abstract

**Background:**

Nephrotoxicity of intravenous (IV) colistin has impeded its clinical use; aerosolized (AS) colistin may be an alternative, but safety data are lacking. Therefore, this study aimed to evaluate the incidence of acute kidney injury (AKI) and risk factors associated with IV and AS colistin administration.

**Methods:**

A retrospective study was performed in a tertiary referral hospital. Data were collected before and after colistin administration between October 2012 and April 2016. Exclusion criteria were as follows: age less than 18 years, previous colistin administration, concurrent use of IV and AS colistin, dialysis before colistin use, and colistin use for less than 3 days. We compared AKI incidence following administration of IV versus AS colistin and analyzed risk factors for colistin-associated nephrotoxicity.

**Results:**

A total of 464 patients were enrolled (n = 311, IV group; n = 153, AS group). Incidence of AKI was significantly higher in the IV group (IV vs AS, 20.26% vs 7.84%, p-value < 0.001). Duration of colistin use (OR 1.033, 95% CI 1.009–1.058, p-value 0.008) and presence of chronic kidney disease (OR 2.710, 95% CI 1.348–5.448, p-value 0.005) were associated with nephrotoxicity. There were no significant risk factors associated with AS colistin.

**Conclusions:**

Although AS colistin was not associated with any significant risk factors for nephrotoxicity, duration of colistin use and baseline kidney function may affect AS colistin-associated nephrotoxicity.

## Introduction

Recently, incidence of infections with multidrug-resistant gram-negative (MDR G (-)) pathogens, such as *Pseudomonas aeruginosa*, *Acinetobacter baumannii*, *Klebsiella pneumoniae*, and *Enterobacter*, has risen. These infections represent a threat to public health because these gram-negative bacteria are resistant to commonly used antibiotics such as antipseudomonal penicillins and cephalosporins, aminoglycosides, tetracyclines, fluoroquinolones, and carbapenems [1]. Elimination of the pathogen is necessary for successful treatment. Therefore, the emergence of MDR G (-) pathogens has encouraged clinicians to review existing antibiotics for alternative treatment options, of which colistin is a potential alternative for treatment of MDR G (-) pathogens [1–3].

Colistin is a mixture of the cyclic polypeptides colistin A and B and is effective against gram-negative bacilli. Colistin was first used clinically in the 1960s. However, use of colistin has declined since the 1970s due to adverse side effects and development of less-toxic antibiotics [4,5]. The major adverse drug reaction resulting from colistin use is nephrotoxicity, which is the main obstacle to its widespread use [5,6]. Many peer-reviewed studies have examined colistin-induced nephrotoxicity.[6–12]. Colistin induces nephrotoxicity through increased tubular epithelial cell membrane permeability. As a result, cations, anions, and water are able to enter cells more freely, leading to cell swelling and lysis. Finally, acute tubular necrosis occurs through increased serum creatinine (SCr) level and decreased creatinine clearance (CLCr) [13]. Nephrotoxicity may be linked to changes in transepithelial conductance in epithelial cells, as demonstrated *in vitro* in rabbit bladder epithelium [13]. The prevalence of the reported nephrotoxicity varied between 10.9% and 45% [6]. We attributed this wide range to the different definitions of nephrotoxicity applied in each study and inclusion of patients with different characteristics [7].

Colistin can be administered via two routes, IV or aerosolized (AS). IV colistin treatment of pulmonary infection is ineffective owing to low penetration of IV colistin into the lung parenchyma [14]. As a result, AS colistin has emerged as an alternative therapy to increase efficacy and avoid systemic toxicity in treatment of lung infections [15,16]. Subsequently, meta-analyses, systemic reviews, and retrospective studies have been conducted to compare the efficacies of IV and AS colistin. These studies reported that efficacy of AS colistin was comparable to IV colistin [17–20]. AS colistin is theoretically regarded to be safer because colistin-associated nephrotoxicity is known to be dose-dependent and associated with tubular necrosis through an increase in tubular epithelial cell membrane permeability; furthermore, large amounts of AS colistin is not delivered to the systemic blood [6,21]. Some studies have shown that nebulized colistin induces less nephrotoxicity than IV colistin. One recent retrospective study demonstrated that nephrotoxicity was less common in AS than in IV colistin monotherapy [20]. In addition, other studies also showed less nephrotoxicity resulting from AS colistin monotherapy. However, nephrotoxicity resulting from AS colistin was not well-characterized because of a small patient sample size [18,19]. Moreover, combination treatment with AS and IV colistin showed similar nephrotoxicity to IV colistin monotherapy [18,22–25]. Based on our review, risk of nephrotoxicity resulting from AS colistin treatment is low. However, factors that may influence nephrotoxicity resulting from AS colistin use are not well-characterized. The aim of this study was to evaluate the incidence of acute kidney injury (AKI) and risk factors of AKI associated with IV or AS colistin monotherapy. This study will provide more complete information regarding factors that should be considered to reduce colistin-associated toxicity.

## Materials and methods

### Subjects

This single-center retrospective study was conducted in Severance Hospital, Seoul, South Korea. In total, 893 patients who were administered IV or AS colistin between October 2012 and April 2016 were evaluated. Subjects who were outpatients, younger than 18 years, had been previously administered colistin, used colistin for less than 3 days, received dialysis before colistin use, and who used IV and AS colistin concurrently were excluded from the study. According to the product label in Korea, 3 million units (MU) equals 100 mg. In Korea, the recommended dose is 2.5–5 mg/kg/d (75,000–150,000 units/kg/d) of IV colistin in 2–4 doses without a loading dose, and dose can be adjusted according to kidney function. Weight is based on actual body weight (ABW) except for obese patients, who use ideal body weight (IBW) for dosing [26]. Different from IV colistin, AS colistin is used as an off-label drug, thus, dosage range varies because clinicians usually rely on their experience to determine the dose. However, 75 mg (2.25 MU) of AS colistin was usually administered 3 to 4 times per day. The study protocol was approved by the institutional review board (IRB) of Yonsei University Health System (Seoul, South Korea, IRB No.4-2016-0490) and all data provided form hospital were anonymized and informed consent was waived by IRB.

### Data collection

The following patient information was gathered from electronic medical records (EMRs): age, sex, actual body weight (ABW), height, colistin administration route, colistin dose, duration of colistin use, SCr before and after colistin administration, blood urea nitrogen (BUN) before and after colistin administration, dialysis record after colistin use, underlying diseases (hypertension, diabetes mellitus, and chronic kidney disease), infected pathogens, APACHE2 (Acute Physiology and Chronic Health Evaluation) score, and other concomitant nephrotoxins [7,27]. If the EMRs contained several data points on the first or last day of colistin administration, the mean value was used; if records were not available on the first or last day of colistin administration, the nearest record was used. APACHE2 score was calculated only in ICU patients.

### Definitions

IBW was calculated by using the Devine formula for height of more than 5 feet (for men, 50 kg + 2.3 kg for each inch over 5 feet; for women, 45.5 kg + 2.3 kg for each inch over 5 feet). IBW was calculated by the equations of less than 5 feet (for men, 50 kg -2.3 kg for each inch less 5 feet; for women, 45.5 kg-2.3kg for each inch less 5 feet [26]. Accumulation dose, daily dose, daily dose per ABW, and daily dose per IBW were calculated to determine if these doses could affect nephrotoxicity.

The estimated glomerular filtration rate (eGFR) was calculated to assess kidney function by using the abbreviated MDRD study equation [eGFR = 175 × SCr^-1.154^ × Age^-0.203^ × Sex (0.742 for female, 1 for male) × Race (1.21 for black, 1 for nonblack)] [28].

Nephrotoxicity by colistin was originally defined using the KDIGO 2012 guideline [29]. However, to reflect clinical situations that SCr is elevated, but kidney function is normal, nephrotoxicity was defined by SCr exceeding 2 mg/dL (176.8 μmol/L) after colistin use when patients’ baseline eGFR was higher than 60 mL/min/1.73 m^2^ [30]. Patients with baseline eGFR less than 60 mL/min/1.73 m^2^, and elevated SCr by more than 1.5 times control, or 0.3 mg/dL, were considered to have colistin-induced nephrotoxicity per KDIGO 2012 guideline [29]; All subjects that underwent dialysis after colistin use were also considered to have nephrotoxicity.

### Statistical analysis

Statistical analyses were performed using SPSS ver. 23 (IBM corporation, Armonk, New York, U.S.). All tests were two-sided and the significance level was set at 0.05 (α = 0.05). Continuous variables were presented as mean and standard deviation (SD), while categorical variables were presented as number (n) and proportion (%). Nephrotoxicity was evaluated based on AKI incidences resulting from IV or AS colistin administration, and the risk factors for IV or AS colistin-associated nephrotoxicity were analyzed. An independent *t*-test was used for continuous variables and Fisher’s exact test was used for categorical variables. Binary multivariate logistic regression was used to evaluate risk factors for colistin-associated nephrotoxicity. Covariates that showed statistical significance in univariate logistic regression were selected for multivariate logistic regression.

## Results

### Patient characteristics

**Fig 1:**
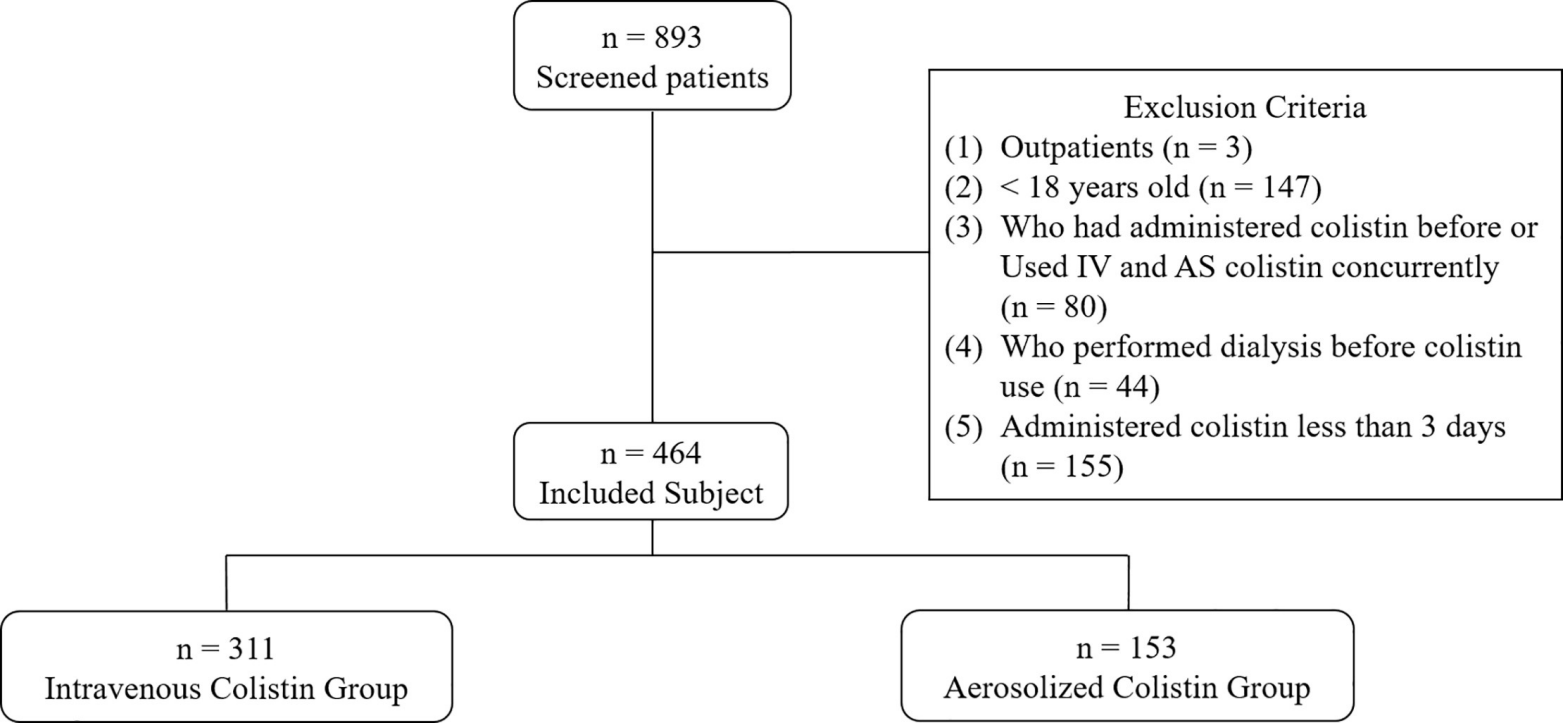
Study design.

A total of 893 subjects were screened. In total, 385 subjects were excluded for the following reasons (Fig 1): outpatient status (n = 3), age less than 18 years (n = 147), previous use of colistin or concurrent use of IV and AS colistin (n = 80), dialysis prior to colistin use (n = 44), and colistin use for less than 3 days (n = 155). Thus, 464 subjects were included in the study and were grouped as follows: 311 subjects were treated with IV colistin and 153 subjects were treated with AS colistin. Colistin was used when other antibiotics were ineffective and culture results were sensitive to colistin. Most patients were in sepsis. The baseline characteristics of the patients treated with IV and AS colistin are summarized in Table 1. Most characteristics were not significantly different except for age, type of pathogen, the number of ICU patients, dosing, and concomitant nephrotoxins. The results showed that patients treated with IV colistin were younger (IV vs AS, 63.42 vs 67.03 years; p-value 0.021), and all categories of colistin doses (accumulated dose, daily dose, daily dose per ABW, and daily dose per IBW) were higher in the IV group. In the case of concomitant nephrotoxins, liposomal amphotericin B (IV vs AS, 9.97% vs 4.58%; p-value 0.049) and vancomycin (IV vs AS, 29.90% vs 14.38%; p-value < 0.001) were more frequently used in the IV group. In contrast, diuretics (IV vs AS, 18.33% vs 34.64%; p-value < 0.001) and tacrolimus (IV vs AS, 5.79% vs 13.07%; p-value 0.011) were more commonly used in the AS group. Cyclosporine was not used in the AS group. APACHE2 score was calculated after ICU admission, and AS colistin was used more in ICU patients than IV colistin (IV vs AS, 43.73% vs 63.40%; p-value < 0.001). However, APACHE2 score did not differ between the two groups.

**Table 1:** Baseline patient’s characteristics.

	IV (n = 311)		AS (n = 153)		p-value^a^
Age (year)	63.42 (16.43)		67.03 (14.37)		0.021
Sex	Man	Woman	Man	Woman	0.302
	198 (63.67)	113 (36.33)	105 (68.63)	48 (31.37)	
Height (cm)	162.72 (9.92)		164.45 (8.56)		0.065
ABW (kg)	60.67 (12.55)		61.89 (11.62)		0.313
IBW (kg)	57.53 (10.44)		59.34 (9.53)		0.070
Duration of colistin use (day)	13.07 (12.93)		12.61 (9.80)		0.702
Baseline SCr (mg/dL)	1.00 (1.03)		0.85 (0.88)		0.116
Baseline eGFR (mL/min/1.73 m^2^)	129.24 (99.90)		142.40 (92.08)		0.172
Baseline BUN (mg/dL)	32.62 (23.30)		29.41 (18.75)		0.111
Underlying diseases (%)					
Hypertension	192 (61.74)		101 (66.01)		0.416
Diabetes mellitus	107 (34.41)		57 (37.25)		0.606
Chronic kidney disease	78 (25.08)		27 (17.65)		0.077
Type of pathogens (%)					0.009
*Acinetobacter baumannii*	146 (46.95)		64 (41.83)		
*Pseudomonas aeruginosa*	76 (24.44)		28 (18.30)		
*Staphylococcus aureus*	50 (16.08)		31 (20.26)		
*Enterococcus*	18 (5.79)		6 (3.92)		
*Klebsiella pneumoniae*	5 (1.61)		3 (1.96)		
Other gram-negative pathogens	14 (4.50)		18 (11.76)		
Unknown	2 (0.64)		3 (1.96)		
ICU patients	136 (43.73)		97 (63.40)		< 0.001
APACHE2 score^b^	24.55 (8.56)		24.49 (8.17)		0.960
Accumulated dose (mg)	4388.03 (4714.22)		3112.85 (3065.82)		0.001
Daily dose (mg)	366.27 (174.77)		240.71 (93.59)		<0.001
Daily dose per ABW (mg/kg/d)	6.30 (3.62)		4.02 (1.74)		<0.001
Daily dose per IBW (mg/kg/d)	6.81 (6.29)		4.19 (1.86)		<0.001
Nephrotoxins (%)					
Amphotericin B (Liposomal)	31 (9.97)		7 (4.58)		0.049
Amphotericin B (Deoxycholate)	46 (14.79)		15 (9.80)		0.146
NSAIDs	79 (25.40)		43 (28.10)		0.575
ACE inhibitors	6 (1.93)		1 (0.65)		0.434
ARBs	21 (6.75)		11 (7.19)		0.848
Vasopressor	228 (73.31)		108 (70.59)		0.581
Diuretics	57 (18.33)		53 (34.64)		<0.001
Aminoglycosides	4 (1.29)		3 (1.96)		0.689
Rifampin	18 (5.79)		10 (6.54)		0.836
Vancomycin	93 (29.90)		22 (14.38)		<0.001
Cyclosporine	5 (1.61)		0 (0.00)		0.176
Tacrolimus	18 (5.79)		20 (13.07)		0.011
Radiocontrast	29 (9.32)		9 (5.88)		0.279

Data are presented as mean and SD for continuous data and number of subjects and proportion for categorical data. Categorical data are sex, underlying diseases, site of infection, type of pathogens, ICU patients, and nephrotoxic drugs. ABW, actual body weight; IBW, ideal body weights; SCr, serum creatinine; BUN, blood urea nitrogen; eGFR, estimated glomerular filtration rate; NSAIDs, non-steroidal anti-inflammatory drugs; ACE inhibitors, acetylcholinesterase inhibitors; ARBs, angiotensin receptor blockers

^a^p-value was calculated by independent t-test for continuous data and Fisher's exact test for categorical data using SPSS ver.23 (IBM corporation, Armonk, New York, U.S.)

^b^APACHE2 score was calculated only in ICU patients.

### Kidney function assessment

**Fig 2:**
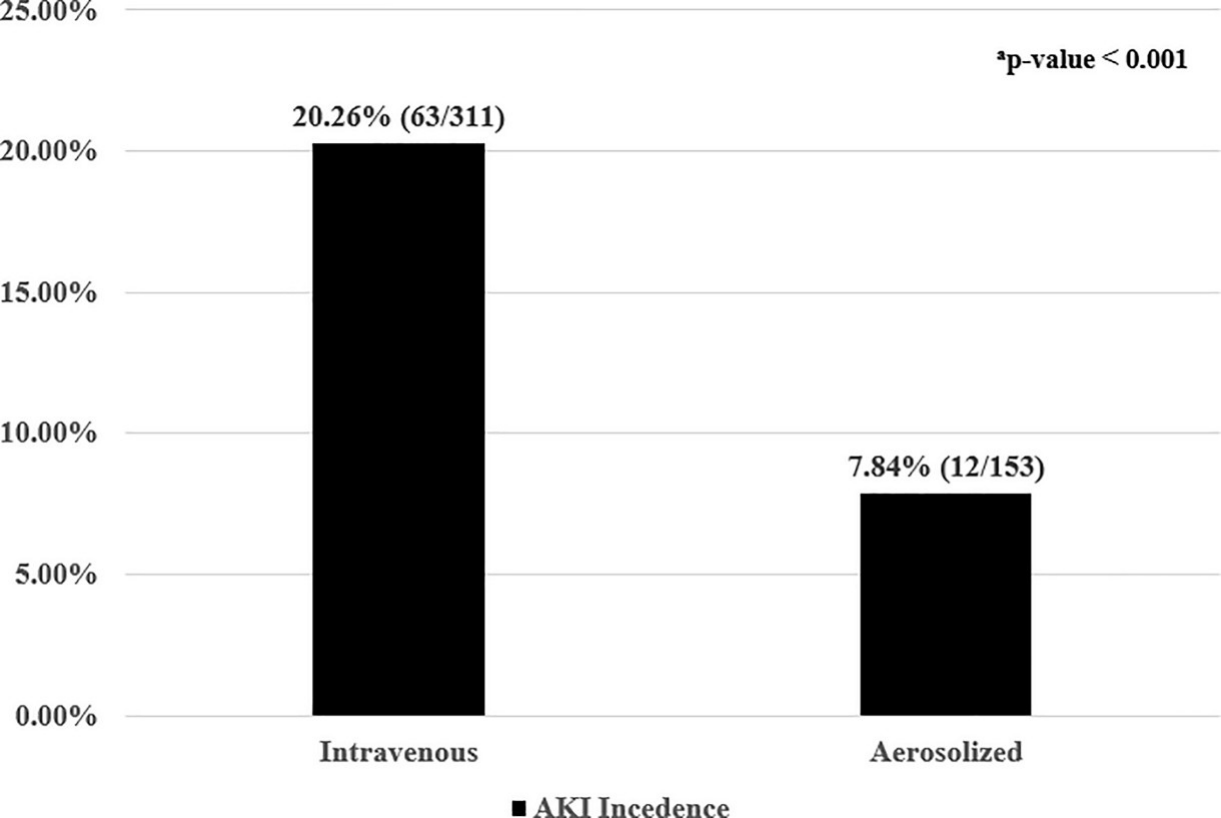
Acute kidney injury incidence due to intravenous (IV) and aerosolized (AS) colistin. Data represent the proportion in each group (number of AKI subjects/total number of subjects); AKI, acute kidney injury; ^a^p-value was lower than 0.001 and was calculated by Fisher's exact test for categorical data using SPSS ver.23 (IBM corporation, Armonk, New York, U.S.).

AKI developed in 63 (20.26%) of the 311 patients in the IV group and 12 (7.84%) of the 153 patients in the AS group (Fig 2). AKI was significantly more frequent in the IV group than in the AS group (p-value < 0.001).

### Risk factors for IV and AS colistin-associated nephrotoxicity

Binary univariate and multivariate logistic regression analyses were performed to determine the risk factors for IV and AS colistin-associated nephrotoxicity (Tables 2 and 3). Age, sex, colistin administration route, height, ABW, IBW, duration of colistin use, baseline SCr, baseline eGFR, baseline BUN, accumulated dose, daily dose, daily dose per ABW, daily dose per IBW, hypertension, diabetes mellitus, chronic kidney disease (CKD), number of nephrotoxins, and each individual nephrotoxin were analyzed as the risk factors.

**Table 2:** Univariate and multivariate analyses of risk factors for intravenous colistin-associated nephrotoxicity.

Characteristics	Univariate analysis		Multivariate analysis	
	OR (95% CI)	p-value^a^	OR (95% CI)	p-value^a^
Age	1.004 (0.987–1.021)	0.676		
Sex	0.530 (0.285–0.987)	0.045		
Height	1.022 (0.992–1.053)	0.149		
ABW	1.021 (0.999–1.044)	0.065		
IBW	1.025 (0.996–1.054)	0.088		
Duration of colistin use	1.039 (1.016–1.062)	0.001	1.033 (1.009–1.058)	0.008
Baseline SCr	1.779 (1.336–2.368)	<0.001		
Baseline eGFR	0.992 (0.988–0.996)	<0.001		
Baseline BUN	1.016 (1.005–1.027)	0.004		
Accumulated dose	1.000 (1.000–1.000)	0.029		
Daily dose	0.998 (0.996–0.999)	0.008		
Daily dose per ABW	0.827 (0.738–0.927)	0.001		
Daily dose per IBW	0.850 (0.766–0.943)	0.002		
Hypertension	2.570 (1.348–4.899)	0.004		
Diabetes mellitus	0.942 (0.524–1.691)	0.841		
Chronic kidney disease	4.533 (2.516–8.166)	<0.001	2.710 (1.348–5.448)	0.005
Number of nephrotoxic drugs	1.180 (0.951–1.463)	0.133		
Amphotericin B (Liposomal)	0.736 (0.271–2.000)	0.548		
Amphotericin B (Deoxycholate)	0.670 (0.284–1.578)	0.359		
NSAIDs	2.159 (1.197–3.896)	0.011		
ACE inhibitors	2.000 (0.358–11.174)	0.430		
ARBs	0.921 (0.299–2.841)	0.886		
Vasopressor	0.981 (0.526–1.831)	0.953		
Diuretics	1.205 (0.604–2.407)	0.596		
Aminoglycosides^b^	-			
Rifampin	0.777 (0.218–2.770)	0.697		
Vancomycin	1.115 (0.614–2.025)	0.721		
Cyclosporine	2.678 (0.438–16.378)	0.286		
Tacrolimus	2.070 (0.745–5.751)	0.163		
Radiocontrast	2.274 (1.000–5.173)	0.050		

OR, odds ratio; CI, confidence interval; ABW, actual body weight; IBW, ideal body weights; SCr, serum creatinine; eGFR, estimated glomerular filtration rate; BUN, blood urea nitrogen; NSAIDs, non-steroidal anti-inflammatory drugs; ACE inhibitors, acetylcholinesterase inhibitors; ARBs, angiotensin receptor blockers

^a^p-value was calculated by logistic regression using SPSS ver.23 (IBM corporation, Armonk, New York, U.S.)

^b^Aminoglycosides was not analyzed due to very low number of cases.

**Table 3:** Univariate and multivariate analyses of risk factors for aerosolized colistin-associated nephrotoxicity.

Characteristics	Univariate analysis		Multivariate analysis	
	OR (95% CI)	p-value^a^	OR (95% CI)	p-value^a^
Age	0.988 (0.950–1.028)	0.552		
Sex	0.711 (0.184–2.753)	0.622		
Height	1.000 (0.934–1.072)	0.992		
ABW	1.024 (0.974–1.076)	0.346		
IBW	1.004 (0.943–1.069)	0.900		
Duration of colistin use	1.047 (1.003–1.094)	0.038		
Baseline SCr	2.723 (1.475–5.026)	0.001		
Baseline eGFR	0.985 (0.973–0.997)	0.011		
Baseline BUN	1.042 (1.016–1.069)	0.001		
Accumulated dose	1.000 (1.000–1.000)	0.070		
Daily dose	1.004 (0.999–1.010)	0.138		
Daily dose per ABW	1.178 (0.871–1.592)	0.287		
Daily dose per IBW	1.205 (0.913–1.590)	0.187		
Hypertension	1.032 (0.296–3.602)	0.960		
Diabetes mellitus	1.223 (0.369–4.049)	0.742		
Chronic kidney disease	1.625 (0.409–6.449)	0.490		
Number of nephrotoxic drugs	1.163 (0.706–1.914)	0.553		
Amphotericin B (Liposomal)	5.440 (0.935–31.654)	0.059		
Amphotericin B (Deoxycholate)	1.969 (0.389–9.969)	0.413		
NSAIDs	0.488 (0.102–2.324)	0.367		
ACE inhibitors^b^	-			
ARBs^b^	-			
Vasopressor	2.194 (0.461–10.440)	0.324		
Diuretics	0.607 (0.157–2.344)	0.469		
Aminoglycosides	6.318 (0.530–75.275)	0.145		
Rifampin	1.333 (0.154–11.511)	0.794		
Vancomycin	1.210 (0.247–5.935)	0.814		
Cyclosporine^b^	-			
Tacrolimus	1.367 (0.277–6.747)	0.701		
Radiocontrast	1.511 (0.173–13.209)	0.709		

OR, odds ratio; CI, confidence interval; ABW, actual body weight; IBW, ideal body weights; SCr, serum creatinine; eGFR, estimated glomerular filtration rate; BUN, blood urea nitrogen; NSAIDs, non-steroidal anti-inflammatory drugs; ACE inhibitors, acetylcholinesterase inhibitors; ARBs, angiotensin receptor blockers

^a^p-value was calculated by logistic regression using SPSS ver.23 (IBM corporation, Armonk, New York, U.S.)

^b^ACE inhibitors, ARBs, and cyclosporine and were not analyzed due to very low number of cases.

In the AS group, there were no significant nephrotoxicity-associated risk factors as determined by multivariate analysis. Angiotensin-converting enzyme (ACE) inhibitors, angiotensin receptor blockers (ARBs), and cyclosporine were not analyzed due to the low number of cases of use concurrent with colistin. However, duration of colistin use, baseline SCr, baseline eGFR, and baseline BUN were determined to be significant risk factors using univariate analysis.

Multivariate analysis of administration of IV colistin showed that longer duration of colistin use and presence of CKD are associated with nephrotoxicity. The odds ratio (OR) of duration of colistin use was 1.033 [95% confidence interval (CI) 1.009–1.058; p-value 0.008], and the OR of CKD was high at 2.710 (95% CI, 1.348–5.448; p-value 0.005).

### Comparison of AKI and non-AKI patients

Between AKI and non-AKI patients, duration of colistin use, kidney function, and accumulated dose were significantly different. In total subjects, the AKI group used colistin for one week longer than the non-AKI group (18.77 ± 18.92 vs 11.79 ± 9.75 days, p-value < 0.001). Factors associated with kidney function were worse in the AKI group (AKI vs non-AKI; baseline SCr, 1.65 ± 1.56 vs 0.82 ± 0.76 mg/dL, p-value < 0.001; baseline eGFR 85.03 ± 75.14 vs 142.94 ± 98.60 mL/min/1.73 m^2^, p-value <0.001; baseline BUN 41.69 ± 27.41 vs 29.61 ± 20.18 mg/dL, p-value < 0.001) and more common in CKD patients [35/75 (46.7%) vs 70/389 (18.0%), p-value <0.001]. There was no difference in the dose between the two groups, but the accumulated dose was higher in the AKI group because colistin was used for a longer duration (5480.71 ± 6049.72 vs 3675.81 ± 3789.46 mg, p-value = 0.015). Comparison of AKI and non-AKI patients treated with AS colistin yielded similar results to the comparison between AKI and non-AKI without AS colistin (Table 4). The comparisons in common were duration of colistin was longer by one week (18.83 ± 8.31 vs 12.09 ± 9.76 days, p-value = 0.022 in AS colistin), kidney function related factors were worse, and accumulated dose was higher in the AKI group treated with AS colistin.

**Table 4:** Comparison of AKI and non-AKI patients treated with AS colistin.

	AKI (n = 12)	Non-AKI (n = 141)	p-value^a^
Duration of colistin use (day)	18.83 (8.31)	12.09 (9.76)	0.022
Baseline SCr (mg/dL)	2.10 (2.11)	0.74 (0.59)	0.047
Baseline eGFR (mL/min/1.73 m^2^)	74.80 (75.62)	148.15 (91.28)	0.008
Baseline BUN (mg/dL)	48.13 (28.53)	27.82 (16.88)	0.032
Chronic kidney disease	3 (25.00)	24 (17.00)	0.445
Accumulated dose (mg)	4783.33 (1810.81)	2970.68 (3112.52)	0.049
Daily dose (mg)	279.81 (113.32)	237.38 (91.42)	0.132
Daily dose per ABW (mg/kg/d)	4.54 (2.41)	3.98 (1.68)	0.285
Daily dose per IBW (mg/kg/d)	4.87 (2.49)	4.13 (1.79)	0.183
APACHE2 score^b^	26.29 (6.92)	24.36 (8.28)	0.550

Data present mean (SD) for continuous data and number of subjects (proportion) for categorical data. Categorical data are chronic kidney disease

^a^p-value was calculated by independent t-test for continuous data and Fisher's exact test for categorical data using SPSS ver.23 (IBM corporation, Armonk, New York, U.S.)

^b^Only ICU patients were calculated APACHE2 score. The number of ICU patients with AKI was 7, and the number of ICU patients with non-AKI was 90.

## Discussion

Use of AS colistin for improved efficacy and reduction of systemic toxicity in pulmonary infection has increased. However, studies of nephrotoxicity resulting from AS colistin treatment are sparse, and those that were reported contained only a small number of patients [15,16,18,19]. In one study, AS colistin monotherapy resulted in less nephrotoxicity than IV colistin monotherapy [20]. However, nephrotoxicity associated factors were not evaluated. In our study, the theoretical safety of AS colistin with regard to nephrotoxicity was evaluated and compared to nephrotoxicity resulting from IV colistin treatment. In addition, we attempted to elucidate risk factors that can affect AS or IV colistin-induced nephrotoxicity.

In this study, we defined nephrotoxicity as follows: SCr elevated more than 1.5 times or 0.3 mg/dL in patients having baseline eGFR less than 60 mL/min/1.73 m^2^ (compared to the control), according to KDIGO 2012 guidelines [29]; SCr exceeding 2 mg/dL (176.8 μmol/L) after colistin use in patients having baseline eGFR higher than 60 mL/min/1.73 m^2^ [30]. We defined patients with nephrotoxicity as having baseline eGFR higher than 60 mL/min/1.73 m^2^ because factors that affect renal function could not be precisely controlled because this was a retrospective study. In addition, we felt that simply increasing the SCr threshold by 50% in patients with normal kidney function would be unlikely to be representative of true nephrotoxicity. Despite a 1.5-times increase in SCr in the normal kidney function group, many patients presented with low Scr. Individuals with low SCr were not considered to have impaired renal function and were not treated in clinical practice. According to these standards, the results of our study demonstrated that nephrotoxicity occurred twice as frequently with IV versus AS colistin treatment (20.26% vs 7.84%; p-value < 0.001, Fig 2). Few studies have compared nephrotoxicity between IV colistin monotherapy and AS colistin monotherapy. One retrospective observational study showed no nephrotoxic adverse events with AS colistin treatment. However, only six patients received AS colistin [18]. In another retrospective study, nephrotoxicity incidence was 4 times higher with IV colistin compared to AS colistin[20]. These studies agree with the results of our retrospective study.

In the multivariate analyses performed to determine risk factors for AS colistin-associated nephrotoxicity, no significant risk factors were identified. However, duration of colistin use, baseline SCr, baseline eGFR, and baseline BUN were associated with nephrotoxicity resulting from AS colistin treatment as determined by univariate analyses (Table 3). Similarly, these factors were also significantly associated with nephrotoxicity in the univariate analysis of IV colistin. This indicates that duration of colistin use and baseline kidney function may be associated with colistin-induced nephrotoxicity.

IV colistin-associated nephrotoxicity risk may increase with duration of colistin use. A second risk factor for IV colistin-associated nephrotoxicity was the presence of CKD. The OR of CKD was high at 2.710 (range, 1.348–5.448), meaning that patients with CKD have a high risk of nephrotoxicity. As such, colistin should be used with caution in CKD patients. Contrary to our findings, previous studies have described old age, longer duration of colistin use, diabetes mellitus, high dose per IBW, and septic shock as risk factors [10,31,32].

Several studies have examined association of IV colistin-associated AKI with nephrotoxins. Use of vancomycin, glycopeptides, NSAIDs, loop diuretics, and rifampin were reported to induce IV colistin-associated AKI [7,10,31–33]. Furthermore, co-administration of more than three nephrotoxins was reported to increase the risk of nephrotoxicity [24]. However, an increase in the number of nephrotoxins did not increase risk in our study. However, univariate analysis showed a significant increase in IV colistin-associated nephrotoxicity with co-administration of NSAIDS. Thus, additional studies on nephrotoxicity resulting from co-administration of NSAIDs and colistin are needed to clarify this relationship.

Several studies have examined dose-dependence of IV colistin-induced nephrotoxicity. Kwon *et al*. [34] reported that dose per IBW was related to nephrotoxicity risk in patients administered IV colistin. Lee *et al*. [7] reported that dose per IBW was a risk factor only in patients with impaired kidney function. Two other studies found that use of more than 5 mg/kg/day (IBW) IV colistin increased nephrotoxicity risk [10,32]. However, dose was not related to colistin-associated nephrotoxicity in our study. Additionally, old age, prolonged colistin administration, hypoalbuminemia, low serum albumin level, high Charlson Comorbidity Index, and the presence of septic shock were reported to be related to nephrotoxicity [7,8,31,32,34].

Comparison of the AKI group and non-AKI group showed that the AKI group used colistin for one week longer. From these results, it could be inferred that duration of colistin use affects nephrotoxicity. Accumulated dose is also significantly higher in the AKI group, but this may be due to the longer use of colistin in AKI. These results showed the same trend with AS colistin administration (Table 4). Colistin is not commonly used in early phases of treatment. Colistin is used when other antibiotics have failed or colistin is suitable based on a pathogen susceptibility test. It is possible that colistin was used in many cases because it was the only alternative. As such, it is possible that colistin was used for long periods of time despite symptoms of nephrotoxicity because there are no other viable options or treatment.

Our study has some limitations that merit discussion. As this was a retrospective study, inaccurate patient information may have been provided. If data were missing, data collected most closely in time to the missing data were used. Furthermore, the mean value was used if there were multiple values obtained for the same factor on the same day. In addition, Patient clinical status was not evaluated using an APACHE II score, which would have been helpful in assessing kidney function and risk of AKI. Despite these limitations, this study, to our knowledge, is the first to attempt to determine risk factors associated AS colistin-induced nephrotoxicity. Furthermore, this is the first study to evaluate nephrotoxicity risks between IV and AS colistin treatment in a large number of patients.

## Conclusion

From the analysis, duration of colistin use and baseline kidney function may be associated with AS colistin-associated nephrotoxicity. Therefore, to reduce nephrotoxicity, short term use of AS colistin and evaluation of baseline kidney function prior to treatment are recommended.

## Supporting information

S1 Dataset Colistin_Data.sav. All data for this study were included and personal information were anonymous.
(SAV)
